# Cross-talk between the microbiome and chronic inflammation in esophageal cancer: potential driver of oncogenesis

**DOI:** 10.1007/s10555-022-10026-6

**Published:** 2022-05-05

**Authors:** Tarang Sharma, Ashna Gupta, Ravi Chauhan, Ajaz A. Bhat, Sabah Nisar, Sheema Hashem, Sabah Akhtar, Aamir Ahmad, Mohammad Haris, Mayank Singh, Shahab Uddin

**Affiliations:** 1 Department of Medical Oncology (Lab), All India Institute of Medical Sciences, New Delhi, India; 2 Laboratory of Molecular and Metabolic Imaging, Cancer Research Department, Sidra Medicine, Doha, Qatar; 3 Translational Research Institute, Academic Health System, Hamad Medical Corporation, Doha, Qatar; 4 Dermatology Institute, Academic Health System, Hamad Medical Corporation, Doha, Qatar; 5 Center for Advanced Metabolic Imaging in Precision Medicine, Department of Radiology, Perelman School of Medicine at the University of Pennsylvania, PA Philadelphia, USA; 6 Laboratory Animal Research Center, Qatar University, Doha, Qatar

**Keywords:** Esophageal squamous cell carcinoma, Esophageal adenocarcinoma, Inflammation, Microbiome, Tumor microenvironment

## Abstract

Esophageal cancer (EC) is frequently considered a lethal malignancy and is often identified at a later stage. It is one of the major causes of cancer-related deaths globally. The conventional treatment methods like chemotherapy, radiotherapy, and surgery offer limited efficacy and poor clinical outcome with a less than 25% 5-year survival rate. The poor prognosis of EC persists despite the growth in the development of diagnostic and therapeutic modalities to treat EC. This underlines the need to elucidate the complex molecular mechanisms that drive esophageal oncogenesis. Apart from the role of the tumor microenvironment and its structural and cellular components in tumorigenesis, mounting evidence points towards the involvement of the esophageal microbiome, inflammation, and their cross-talk in promoting esophageal cancer. The current review summarizes recent research that delineates the underlying molecular mechanisms by which the microbiota and inflammation promote the pathophysiology of esophageal cancer, thus unraveling targets for potential therapeutic intervention.

## Introduction

Esophageal cancer (EC) is the sixth most common cause of cancer mortality and the eighth-most commonly detected cancer worldwide [1]. Histologically, esophageal squamous cell carcinoma (ESCC) is the most predominant subtype, followed by the esophageal adenocarcinoma (EAC), the latter being increasingly reported in the western nations [2]. In contrast, ESCC is more common in Asia and Africa [3]. ESCC can be found throughout the esophagus, while EAC occurs in the distal region of the esophagus [3]. Risk factors for EAC are markedly different from those of ESCC. Tobacco smoking, genetic factors, excessive consumption of alcohol, intake of red meat, and very hot beverages are common risk factors for ESCC [4–7]. Another potential risk factor is poor oral health due to the shift in the oral microbiome [8–10]. In comparison, the main risk factors for EAC include obesity, gastroesophageal reflux disease (GERD), *Helicobacter pylori* infection (inverse association), and the pattern of sex difference [11].

Environmental exposure can contribute to chronic inflammation and epithelial cell transformation in both the EC subtypes, leading to precancerous lesions and cancerous tissues. For example, the development of EAC involves chronic exposure of the distal epithelium to stomach and bile acids, triggering inflammation and intestinal metaplasia, also known as Barret’s esophagus (BE) [12]. The esophageal injury can directly be caused by reflux [13] or indirectly by the generation of reactive oxygen species (ROS) [14, 15]. Furthermore, chronic tobacco exposure can also cause tissue damage and inflammation. Chronic irritation of the esophageal epithelium by smoking and alcohol intake is also known to trigger esophageal squamous dysplasia and ESCC via direct toxicity and ROS production [16, 17]. Reportedly, the dysbiosis of oral microbiota has been associated locally with esophageal cancer [18]. One of the major environmental factors known to influence orodental pathophysiology is smoking [19]. The mechanisms by which the toxic components and bacteria in a cigarette can impact oral bacteria include immunosuppression, biofilm formation, and lack of oxygen. These factors could directly or indirectly lead to colonization of harmful bacteria and loss of beneficial bacteria and, consequently, lead to the emergence of diseases, including cancer [20, 21]. Recent studies have uncovered a mechanism by which nicotine addiction induces lymphatic metastasis of esophageal cancer [22]. Mechanistically it indicated that OTU domain-containing protein 3 (OTUD3) is downregulated by nicotine in heavy smokers and correlates with poor prognosis in EC patients. Furthermore, it was shown that downregulation of OTUD3 and its interacting partner, ZFP36, is essential for nicotine-induced VEGF-C production and lymphatic metastasis in esophageal cancer. Overall, this study established that induction of VEGF-C mRNA decay might be a potential therapeutic strategy in EC [22]. Moreover, recent studies suggest that a dysbiotic microbiome may play a significant role in disrupting the epithelial barrier, causing chronic inflammation, and inducing DNA damage and gastrointestinal (GI) carcinogenesis [23]. Although the direct role of esophageal microbiota in EC has not been well defined, growing evidence indicates that significant alterations in commensal flora may play a potential role as a co-factor in the pathogenesis of metaplasia and dysplasia. Reportedly, a shift in the esophageal microbiome towards the gram-negative bacteria leads to LPS mediated inflammation via Toll-like receptor-4 (TLR-4), NF-κB activation, and increased reflux through nitric oxide synthase (NOS) induced relaxation of the esophageal sphincter [24–26]. The poor prognosis and low median survival of both ESCC and EAC are attributed to late symptoms and clinical diagnosis at locally advanced or already metastasized stages.

Standard therapy is limited to surgical or endoscopic resection and chemotherapy. The potential effect of targeted therapy on the prognosis of EC is still unclear, and options are limited to targeting the human epidermal growth factor receptor 2 (HER2) [27, 28], epidermal growth factor receptor (EGFR) [29], or phosphoinositide 3-kinase/mammalian target of rapamycin (PI3K/mTOR) [30]. The addition of specific EGFR or HER2 monoclonal antibodies to concurrent chemoradiation therapy did not exhibit any significant clinical response [26, 27]. Thus, only narrow survival advantages have been observed in EC patients with chemotherapy, surgery, CT, radiation therapy, or targeted therapy. Conceivably, to develop new targeted therapies complementing conventional therapy, the complex molecular mechanisms underlying the pathophysiology of ESCC and EAC need to be deciphered. The progress in high-throughput metagenomic DNA sequencing in recent years has greatly improved our understanding of not only the complex human microbiome but also its potential role in carcinogenesis directly through the adaptive and innate immune system or indirectly through metabolites and toxins in EC [31]. Emerging evidence also indicates that the microbiota can be manipulated to treat several diseases like cancer; consequently, a deeper understanding of how the microbiome-immune system cross-talk contributes to EC tumorigenesis can guide the development of future therapeutics and diagnostics for EC [32, 33].

Here, we review recent studies that demonstrate the potential association between EC, chronic inflammation, and microbiome in the gut and esophagus. We also summarize chronic inflammatory microbiota-mediated pathways as potential targets for EC therapy.

## Tumor microenvironment in the progression of EC

The esophageal cancer tumor microenvironment (TME) is a very dynamic and complex network of cell types such as immune cells, endothelial cells, cancer-associated fibroblasts (CAFs), adipocytes, extracellular matrix proteins like fibronectins, collagen, elastin, proteoglycans, and hyaluronic acid, and secretory proteins like chemokines, cytokines, and growth factors [34]. Furthermore, there is infiltration of TME with tumor-associated macrophages (TAMs), regulatory T (Treg) cells, myeloid-derived suppressor cells (MDSCs), which are immunosuppressive in their function [35]. The chemokines, growth factors, and cytokines secreted by the tumor cells further induce the immune cells to reprogram the TME, thereby promoting tumorigenesis, metastasis, and resistance to chemoradiation therapy (CRT) [36–39]. Since the TME components play a major role in modulating the CRT response and inducing resistance, they can be exploited as potential targets for therapy [40, 41]. For example, immune cell-secreted cytokines and chemokines activate several downstream effector pathways such as JAK/STAT and NF-κB, which induce several hallmarks of tumorigenesis [42]. Notably, in patients undergoing esophagectomy, the IL-6 and IL-6Rα induced activation of STAT3 has been associated with poor prognosis. However, robust clinical studies are yet to be carried out in this area. Conversely, the inhibition of STAT3 has shown potential in EC in preclinical studies [43]. NF-κB is an important transcription factor that regulates the expression of genes involved in the immune/inflammatory responses and cell proliferation. NF-κB modulates EC TME and might be a potential molecular target in ESCC as it is selectively expressed in this type of cancer, and its expression is linked to poor prognosis and resistance to CRT in ESCC patients [44]. It constitutes a key mechanism linked with inflammation, metastases, and poor prognosis in EC patients [45]. An effective mechanism by which chronic inflammation causes EC involves the cross-talk between STAT3 and NF-κB signaling pathways by modulating EC TME [39]. Moreover, the stabilization of the transcription factor, hypoxia-inducible factor-1α (HIF-1α), in the tumor core upregulates several pro-angiogenic genes expressing cytokines and growth factors that subsequently induce an angiogenic response. One of the most potent pro-angiogenic factors mediating cell survival, proliferation, and migration, is the vascular endothelial growth factor (VEGF) in the EC TME [46, 47]. Reportedly, high serum-VEGF levels are associated with tumor progression, poor treatment outcomes, and poor survival in ESCC patients [48]. Notably, chronic inflammation, TME, and angiogenesis in tumour development have a direct role in tumor development, which will be discussed in the subsequent section.

Furthermore, EAC is reported to be triggered by chronic inflammation as a result of GERD leading to metaplasia and upregulation of inflammatory cytokines, and the malignant progression is significantly influenced by immune cells [49, 50]. Studies suggest that tumor-infiltrating macrophages, in both EAC and ESCC, play a critical role in malignant progression and resistance to therapy, thereby acting as a potentially valuable therapeutic target in EC [50]. Although the difficulty with sampling methods and the dynamic esophageal environment limits our present knowledge of the esophageal microbiome, which forms an essential part of EC TME. However, studies report that EC may also be promoted through an imbalance of microbiota, where lack of microbial diversity is seen to be linked with esophageal squamous dysplasia [51, 52]. For instance, the bacteria *Fusobacterium nucleatum*, found in the EC tissue, is linked with shorter survival and is involved in aggressive tumor progression by activating chemokines like CCL20 [53, 54]. Similarly, in patients with high-grade dysplasia and EAC, alterations in the esophageal microbiome have been observed to occur with the progression from BE to EAC, with an increase in *Enterobacteriaceae* and *Akkermansia muciniphila* and reduction of *Veillonella* species [52]*.* Such alterations in the microbiome are implicated to be the cause of a sudden rise in the incidence of EC in the past few decades. Collectively, all the evidence suggests a complex and dynamic network of immune cells, fibroblasts, inflammatory factors, and microbiota have a dominant role in carcinogenesis and the therapeutic outcome of EC.

## Microbiota as a modulator of TME in EC

### Healthy esophageal microbiome

The conventional bacterial culture-based studies have identified only a few esophageal microbial species, such as *Streptococcus viridans*, expelled from the stomach by reflux or swallowed from the oropharynx [55, 56]. However, most indigenous oesophagal microflora is non-culturable and likely to go undetected by traditional culturing methods. Therefore, a more advanced methodology such as polymerase chain reaction (PCR) of 16S ribosomal RNA has been used to characterize the native esophageal microbiota delineating 95 species under 6 phyla, namely Firmicutes, Bacteroides, Proteobacteria, Fusobacteria, Actinobacteria, and TM7 [55, 57]. Microscopic examination of the tissue revealed a close link between the bacteria and the mucosal epithelium, indicating a stable residential flora. Another study through 16S rRNA sequencing characterized the distal esophageal microbiome in healthy patients, thus revealing the presence of 9 phyla and 166 species, predominantly *Streptococcus* [24]. Similarly, another published study confirmed the predominant esophageal bacterial flora of *Streptococcus*, *Prevotella*, and *Veillonella* through a minimally invasive esophageal string test in 15 pediatric patients, and the string samples from esophagus were analyzed by rRNA gene sequencing [58]. These studies collectively show that bacterial taxa, namely *Streptococcus*, *Haemophilus*, *Neisseria*, *Prevotella*, and *Veillonella,* most commonly inhabit the normal esophagus.

Intriguingly, even in the normal esophagus, the microflora composition may vary according to different factors, one of them being age. In the esophageal microbiome, age was found to be positively correlated with the prevalence of *Streptococcus* species [59]. Although the effect of age on the composition of the gastric microbiome has been studied [60], but its influence on the esophageal microbiome is not yet understood. Findings suggest that the composition of stomach microflora may change due to chronic inflammation and decreased acidity by aging [61], which could modulate the esophageal microbiome since gastric content can influence esophageal mucosa. An additional factor that can influence the esophageal microbiome is the intake of proton pump inhibitors (PPIs), with studies demonstrating dramatic changes in the esophageal tissue microbiota in esophagitis patients due to PPI treatment [62]. PPIs are believed to modulate the esophageal microbiome by increasing the gastric pH and decreasing the acid exposure in the mucosa of the distal esophagus [63] or by directly targeting the proton pumps of specific bacteria like *H. pylori.*

Moreover, the esophageal microbiome can also be altered by diet, as a high dietary fiber intake was reportedly linked with an increased number of Firmicutes and a decreased number of gram-negative bacteria. In contrast, a low fiber intake was associated with an increased number of gram-negative bacteria, like *Parvimonas* and *Eikenella* [64]. The microbial diversity and the presence of potentially pathogenic genera like *Parvimonas* and *Porphyromonas* are positively correlated with poor periodontal health [51]. Collectively these findings indicate that the esophageal microbiome is susceptible to lifestyle and environmental factors.

### Microbiota in EC

The microbial population in the normal healthy esophageal tissue differs markedly from that in pathological conditions like the BE, GERD, and EAC. One of the foremost risk factors of EAC, GERD, also influences the esophageal microbiome, with *Firmicutes and Proteobacteria* being the predominating phyla in the esophageal microbiome of GERD patients [24, 59, 65–68]. Reportedly, both BE and GERD patients demonstrate an increase in gram-negative bacteria such as *Prevotella, Neisseria, Campylobacter, Leptotrichia,* and *Fusobacterium* and a decrease in gram-positive bacteria composition [59, 65–68]. Although limited in their sample size, studies have demonstrated a shift in esophageal microbiome in patients with a milder form of GERD, called non-erosive reflux disease (NERD), where an increase in *Bacteroidetes and Proteobacteria* and decrease in *Fusobacteria and Actinobacteria*, was seen with respect to controls [67].

Comparable to GERD, gram-negative bacteria are predominant in the esophageal microbiome of BE patients. Notably, *Campylobacter*, including *Leptotrichia*, *Fusobacterium*, *Rothia*, and *Capnocytophaga*, were found enriched in 29(n) subjects with GERD, 7 with glandular mucosa, and 5 with BE, when compared with 59 control subjects with the histologically normal esophagus [59]. Chronic exposure of the esophageal squamous epithelium to gastric acid and bile salts in the refluxate are thought to cause inflammation and injury, and such changes are likely to contribute to shifts in the BE microbiome [59]. Notably, the microbiome of the metaplastic tissue was found to be different from normal esophageal tissue in BE patients [57], with the lower relative abundance of *Bacteroidetes* and *TM7* at the phylum level and *Prevotella*, *Selenomonas*, *Campylobacter*, and *Fusobacterium,* at the genus level [69]. In another study, the differences in relative abundances of bacterial taxa were observed in esophageal biopsies from proximal, mid, and distal regions [70]. Despite being limited to small sample sizes and cross-sectional analysis, existing studies on the BE microbiome have revealed consistent conclusions, such as alterations in the ratio of *Streptococcus* and *Prevotella*, and the association of gram-negative bacteria. However, prospective studies on large sample size are needed to substantiate that the gram-negative bacteria and the *Streptococcus*: *Prevotella* ratio changes are associated with the etiology of BE.

Since an increasing number of studies suggest that the gut microbiome may play an important role in cancer, an improved understanding of the microbiome in esophageal carcinoma is very critical. Recent studies have utilized 16S sequencing technology to characterize normal and cancerous esophageal tissue microbiota. The microbiota in both EC subtypes was consistently dominated by the oral periodontopathic spirochete like *Treponema denticola*, *Streptococcus mitis*, and *Streptococcus anginosus* [71]. Reportedly, *Campylobacters* were significantly enriched in GERD and BE than in the controls and EAC, and tissues colonized by *Campylobacter* expressed higher levels of IL-18, cytokine linked with carcinogenesis [68]. Studies point towards the pathogenicity of *Campylobacter* species which indicates its role in EAC progression could be similar to that of *H.pylori* in gastric cancer [72].

Interestingly, a study by Sawada et al. found no effect on the incidence of esophageal adenocarcinoma due to antibiotic alteration in the esophageal microbiome [73]. Furthermore, EAC samples show no stable bacterial composition. In contrast, other studies demonstrate a decreased microbial diversity, and another reported increased diversity in EC patients [52, 74]. As a result, there is no consensus regarding the microbial diversity in the EAC microenvironment, and further evidence is needed to arrive at the unanimity. Studies reported an abundance of lactic acid-producing bacteria such as *Staphylococcus*, *Lactobacillus*, *Bifidobacterium*, and *Streptococcus* in the EAC cascade microenvironment, which may mediate carcinogenesis through dysregulated lactate metabolism. However, more studies characterizing the EAC microbiome in a large sample population are required to ascertain the clinical utility of these findings. Nevertheless, these findings suggest that the changes in the microbiome could be potentially responsible for the progression of GERD and BE towards adenocarcinoma.

**Fig. 1:**
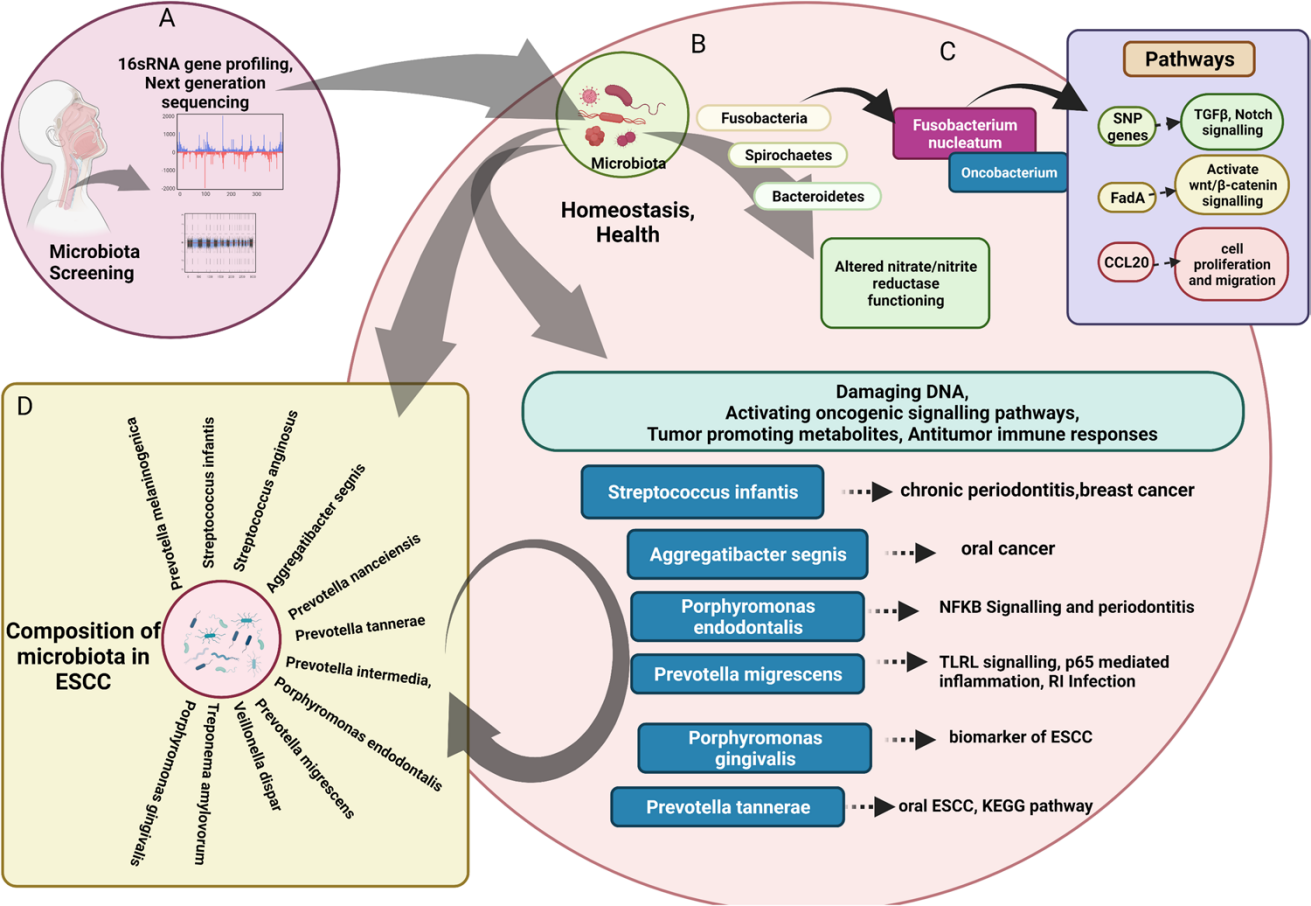
**Esophageal** **microbiota and its influence on inflammatory and oncogenic pathways, which stimulates oncogenesis and progression to ESCC. (A)** Characterization of esohageal micribiome by 16S rRNA sequencing. **(B)** The native microbial phyla composition in the esophagus **(C)** *Fusobacterium nucleatum* in the EC tissue and its association with shorter survival and tumor progression by activating chemokines (CCL2), and beta-catenin pathway through interaction between E-cadherin and FadA, and differential regulation of inflammatory oncogenic pathways. **(D)** High throughput profiling of the esophageal mucosal microbiota in ESCC, delineating top 10 bacteria strains that may influence carcinogenesis and their role in other types of cancer

Through 16S rDNA sequencing technology, it has been shown that gastric dysbiosis is involved in the progression from esophageal squamous dysplasia to squamous cell carcinoma (Fig. 1A) [75]. Reportedly, *Porphyromonas gingivalis* was found to infect esophageal mucosa of ESCC, suggesting a pathogenic role of this microbe in ESCC [76] (Fig. 1). Importantly, its presence in cancer tissue was associated with a significantly shorter survival time. A prospective and high-throughput profiling of the esophageal mucosal microbiota in ESCC was carried out by Yang et al., who have characterized top 10 bacteria strains that may influence carcinogenesis and progression of ESCC. These are namely, *Aggregatibacter segnis*, involved in oral cancer; *Treponema amylovorum*, implicated in chronic periodontitis; *Porphyromonas endodontalis* and *Streptococcus infantis* found in the salivary microbiome and in breast cancer; *Veillonella dispar*, in oral mucosa, which is involved in autoimmune hepatitis; *Streptococcus anginosus*, found to be enriched in gastric cancer; *Prevotella intermedia* and *Prevotella melaninogenica* which cause periodontitis; and *Prevotella nigrescens*, which induces inflammation and also causes respiratory tract infections [77] (Fig. 1B,C). Nevertheless, more studies are required to validate these findings and delineate the mechanism by which alteration in the microbiome leads to the development of esophageal carcinoma.

Viruses along with bacteria are an important component of microbiota. Viruses have been demonstrated to be a stable component of the total microbial ecosystem of the gastrointestinal tract (the esophagus, stomach, and colon) [78]. Notably, viral infections may play a role in carcinogenesis and the development of tumors by modulating the immune homeostasis and inducing DNA alterations through viral-dependent mechanisms [79]. Mounting evidence indicated that human papillomavirus (HPV) is one of the important viral pathogens that can lead to colon carcinogenesis [80, 81]. Furthermore, Epstein–Barr virus (EBV) is known to be an important etiological agent of gastric cancer (GC), which is characterized by unique genomic aberrations and pathological features. Post-infection, EBV integrates its DNA into the host and expresses latent protein, and impairs DNA methylation via miRNA, leading to EBV-positive gastric cancer [82]. Notably, HPV and EBV infections are reported to increase the risk of esophageal squamous cell carcinoma (ESCC) [83]. Furthermore, viruses, like bacteriophages, have also been detected in the esophageal microbiome and could target bacteria [59]. The association between the esophageal virome and adenocarcinoma is an active research area and is the focus of many studies. Many questions still remain unanswered in this context like, Can eukaryotic viruses trigger carcinogenesis by inducing mutational events that transform the epithelial cells leading to tumorigenesis? Future studies of the association of virome with EC will answer these basic questions.

### How esophageal microbiome contributes to EC

Overall, EC is associated with the bacterial profile, which most likely activates the innate immune system. The gram-negative bacteria found in BE produce lipopolysaccharide (LPS) that is known to activate innate immune responses by stimulating TLR-4 in the epithelial or immune cells leading to the activation of NF-κB. The increased NF-κB activation is linked to high levels of pro-inflammatory cytokines. This activation of the NF-κB pathway is paralleled by a simultaneous increase in interleukin (IL) IL-1β, IL-6, IL-8, and tumor necrosis factor (TNF) along the spectrum of reflux esophagitis, BE, and adenocarcinoma [84–86]. Given that *Fusobacterium nucleatum* may lead to the development of colorectal cancer, it may also play a role in EC. The study further reported that nearly 23% of EC patients contained *Fusobacterium nucleatum* in their cancer tissue. *F. nucleatum* can activate the beta-catenin pathway through interaction between E-cadherin and *F. nucleatum*–produced FadA adhesion and differentially regulates inflammatory oncogenic pathways [53, 87].

Altogether, the alteration in the microbiome of BE may lead to EAC by triggering chronic inflammation [25]. Another study has reported the prevalence of *Escherichia coli (E.coli)* in BE and EAC with upregulation of TLR 1–3, 6, 7, and 9 in EAC when compared with normal epithelium., pointing out that the initial molecular changes could be induced by microbes as shown in the rat model of EAC [88]. This suggests a possible link between the TLR signaling pathway and *E.coli.*

One of the most extensively studied TLR in the EAC oncogenic cascade is TLR-4 [89, 90]. An increase in TLR-4 expression during malignant changes of esophageal columnar epithelium leads to unfavorable disease outcomes. Interestingly, instead of being confined to the basal layer in the squamous epithelium, during reflux, there may be superficial damage to the esophageal epithelium; as a result, there is not only an increased TLR-4 expression but also an increased exposure of the TLR-4 to pathogen-associated molecular patterns (PAMPs). Reportedly, TLR-4 expression was significantly upregulated in EAC, BE, and esophagitis compared to the normal squamous esophageal samples [91]. The stimulation of TLR-4 induces inflammatory response and cyclooxygenase-2 (COX-2) expression in BE. COX-2 is believed to play an essential role in EAC progression in BE [92]. Furthermore, it was found that inhibition of COX-2 prevents adenocarcinoma provoked by reflux. This provides proof that COX-2 inhibitors may have a chemotherapeutic effect in BE. TLR-4 activation in BE may contribute to malignant transformation through the induction of COX-2. One of the mechanisms for the strong increase in COX-2 expression upon TLR-4 activation could be an increased stability / or transcription of COX-2 through NF-κB independent mitogen and stress-activated protein kinase (MSK), mitogen-activated protein kinase (MAPK) pathways [93, 94]. Therefore, the shift in microbiota in the EAC cascade may cause LPS activation of TLR-4 and lead to activation of NF-κB, secretion of IL-8, increased COX-2 expression, and increased proliferation [91]; each of these factors may induce carcinogenesis (Fig. 3). Secondly, COX-2 can induce cell proliferation, inhibition of apoptosis, angiogenesis, and tumor invasiveness [92]. Furthermore, the cytokine IL-8 may play a role in carcinogenesis through the regulation of angiogenesis, cancer cell growth, tumor cell movement, leukocyte infiltration, and immune responses [95]. As a result, the shift in BE microbiota may induce the carcinogenesis of the EAC cascade through the activation of TLR-4.

**Fig. 2:**
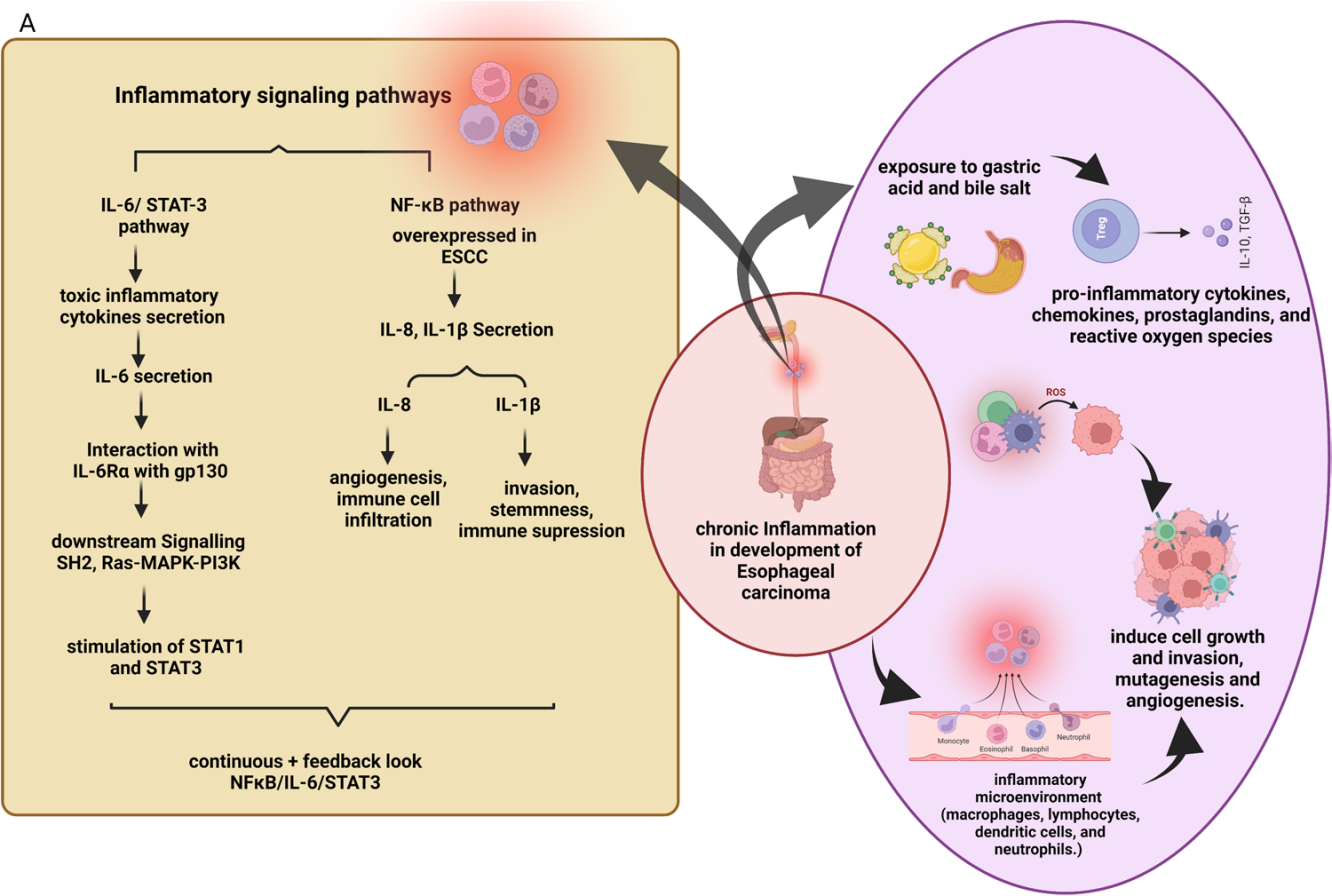
**Chronic inflammatory pathways associated with ESCC and their role in aiding carcinogenesis in ESCC. (A)** Downstream inflammatory signaling pathways (IL-6/STAT3, NF-kB) implicated in the development and progression of esophageal carcinogenesis **(B)** Induction of tumor cell growth, invasion, and angiogenesis by chronic exposure to gastric acid and bile salts and the role of inflammatory microenvironment in the development of esophageal carcinoma

**Fig. 3:**
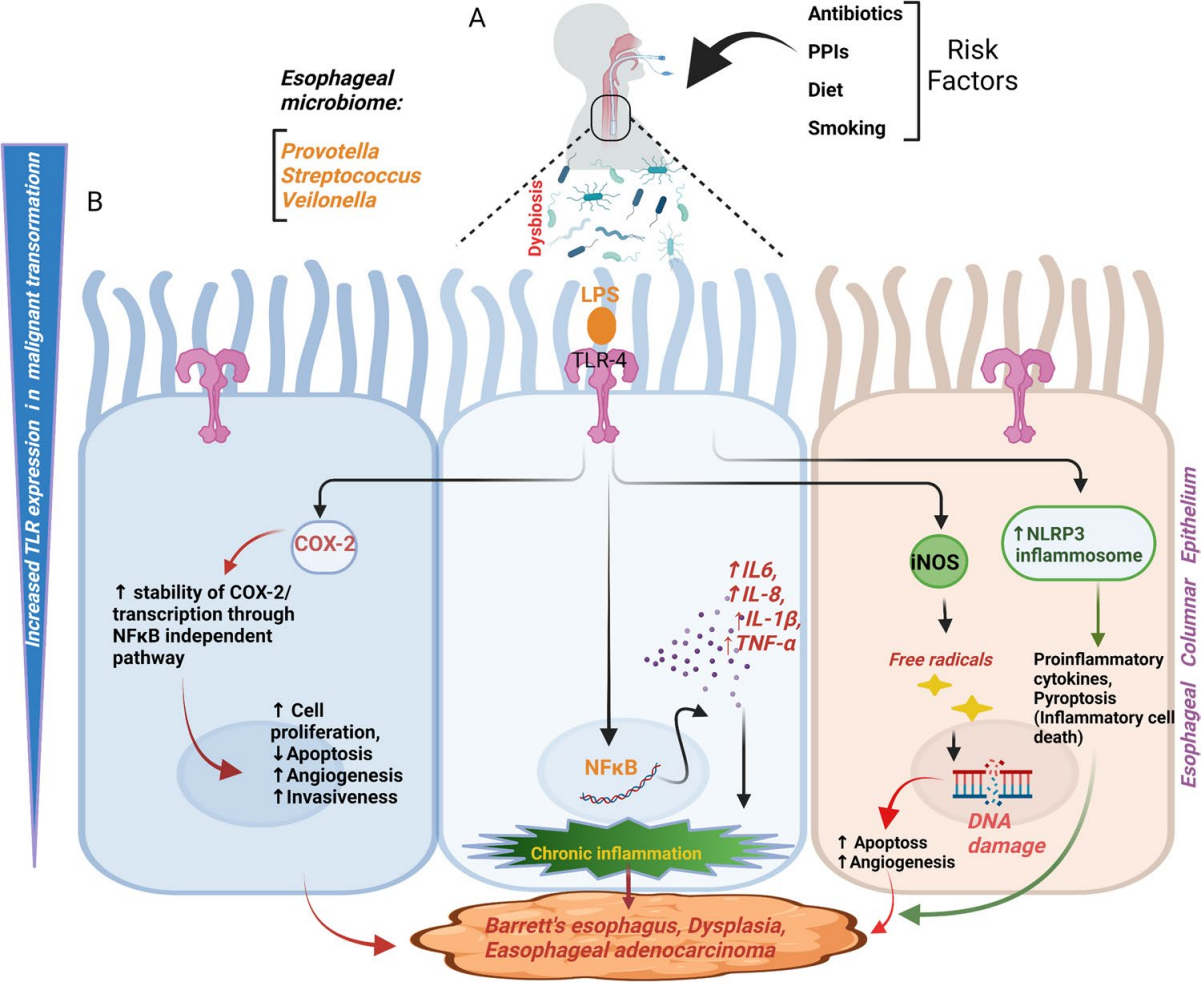
**Dysbiosis-associated pathways and their role in promoting EC. (A)** The risk factors associated with microbial dysbiosis and esophageal tumorigenesis; and the predominant component of a healthy esophageal microbiome. **(B)** Increased TLR expression during esophageal malignant transformation and induction of downstream signaling pathways (COX-2, NLRP inflammasome, iNOS, NF-kB) upon TLR activation by PAMPs, and the consequent development and progression of EC

TLR expression is intricately connected to esophageal microbiota, with research studies showing the variable location of Toll-like receptor-2 (TLR-2) expression in EAC. In BE, TLR-2 was expressed in normal oesophagal epithelium basal keratinocytes and the superficial epithelial cells and lamina propria [96]. However, in EAC, there was diffused TLR2 expression throughout the biopsy. Long-term activation of TLR-2 in bile salts exposed BE epithelium cells (BAR-T) results in more expression of mitochondrial enzymes, lysosomal enzymes, and other factors involved in endocytosis. [96]. Furthermore, TLR-2, due to its ability to heterodimerize with other TLRs and its ability to recognize a wide range of ligands, may play an important role in identifying dysbiotic microbial components. Studies show that TLR2/6 and TLR1/6 heterodimers recognize several PAMPs of dysbiotic microbiota, including bacterial cell wall components. *Huhta* et al*.* suggested heterodimerization, in the EAC cascade, through immunohistochemical analysis of TLR expression in pathologic esophageal samples [89]. They have shown an upregulation of TLR1/2/6-network in Barrett's metaplasia and cancer. Recognition of bacteria by a precancerous metaplastic cell is increased when there is upregulation of TLR1/2/6-network leading to inflammation. (Fig. 2 and 3). Although the microbiota regulates TLR-2 expression in the small intestine, more studies are needed to ascertain if TLR-2 expression directly varies with the composition of the esophageal microbiota. It has been shown that increased cytoplasmic and nuclear expression of TLR-4 is associated with poor prognosis in EAC. Additionally, in EAC tumors, expression of Toll-like receptor-9 (TLR-9) is correlated with high pathological tumor stage, distant organ metastases, high tumor grade, and decreased 10-year survival rates [97]. Many ligands have been described as ligands for TLR-9 such as apoptotic DNA, microbial DNA, and CpG-containing oligonucleotides. Stimulation by these ligands induces invasion in LR-9-expressing cancer cells.

Furthermore, the gram-negative, dysbiotic microbiota in the EAC may stimulate inducible Nitric Oxide Synthase (iNOS), thereby relaxing the lower esophageal sphincter and inducing GERD (Fig. 3). Generally, cells do not express iNOS, but LPS and cytokines can stimulate its expression. High concentrations of NO produced by iNOS can generate free radicals, causing DNA damage and inhibiting DNA repair enzymes [98]. NO, and iNOS activity has been shown to induce apoptosis, angiogenesis, and DNA damage during tumorigenesis [99] (Fig. 3). Clemons et al. have explored the mechanism behind NO-mediated invasion of BE cells and found that NO increases the expression of matrix metalloproteinase (MMP) and its inhibitor (TIMP), and the latter promotes progression of dysplastic lesion of BE to invasive carcinoma [100]. It was further seen that there is a higher expression of iNOS in BE and EAC than in the normal esophagus. In immunohistochemical analysis, it was seen that iNOS expression was high in BE patients compared to matched gastric control [101].

Interestingly, the dysbiotic microbiota in the EAC can induce inflammation-induced carcinogenesis. The nod-like receptor protein 3 (NLRP3), an important component of the innate immune system, mediates the secretion of cytokines IL-1β/IL-18 and caspase-1 activation in response to microbial infection. Various mechanisms have been proposed for NLRP3 mediated inflammasome activation (Fig. 3). The first signal is provided by microbial stimuli or cytokines that activate toll-like receptors or tumor necrosis factor receptors (TNFRs). Consequently, NF-κB is activated by TLRs or TNFRs, thereby causing the upregulation of pro-IL-1β and NLRP3 transcription. The second signal is generated by particulate matter, pore-forming toxins that activate the NLRP3 inflammasome [102]. Studies have shown that the NLRP3 inflammasome may also modulate the esophageal microbiome composition. It has been shown that impaired crypt bactericidal activity in NLRP3 knockout mice has reduced TGF-β and IL-10, an anti-inflammatory cytokine. Also, NLRP3^−/−^mice has decreased colonic antimicrobial secretions that exhibit remarkable changes in intestinal microbiota composition [103]. On the other hand, hyperactive NLRP3, i.e. Nlrp3 R258W mutation, enhances the relationship between gut microbiota and the immune system by remodeling gut microbiota. *NLRP3* ^R258W^ mutation allows Tregs to maintain homeostasis in the gut by enhancing the secretion of IL-1β, an anti-inflammatory cytokine, thus neutralizing the effect of inflammation [104]. The role of dysbiosis on NLRP3 inflammasome activation with respect to EAC has been investigated by treating the normal esophageal squamous cells and BE epithelial cells with LPS in the presence or absence of TLR4 or NLRP3 inflammasome inhibition. It was seen that the LPS activates the NLRP3 inflammasome by inducing the expression of NLRP3, pro-IL1β, and pro-IL18 downstream of TLR4. Secondly, LPS increases mitochondrial ROS that activates the NLRP3 inflammasome. The activated NLRP3 inflammasome subsequently enables pyroptosis and converts pro-IL1β and pro-IL18 into mature IL1β and IL18 [102]. Anecdotal evidence indicates that another important signaling mediator, lactate, may have a role in the oncogenesis of EAC. Lactate is an essential signaling molecule for cancer metabolism and plays an important role in angiogenesis, immune evasion, cell migration, and metastasis [105]. There is a marked lowering of blood glucose levels in cancers with an increase in lactate levels; this is known as the “Warburg effect. The increased glucose uptake by cancer cells and its fermentation into lactate enable their survival and proliferation. In EAC, the microbiota may induce the Warburg effect. Deshpande et al., upon analyzing 106 microbial brush samples, found that GERD or BE esophagus exhibited an upregulated lactate production [59]. Another group showed that the dysbiotic microbiota in EAC consisted of a larger number of lactic acid-producing bacteria, such as *Staphylococcus, Lactobacillus, Bifidobacterium, and Streptococcus* [67] (Fig. 1). However, the exact role of the microbiota on lactate availability and its effect on the host cells needs to be investigated since a comparable increase in lactate-producing bacteria is reported in gastric adenocarcinoma [106]. We have summarized how Esophageal microbiota influences different inflammatory and oncogenic pathways, which stimulates oncogenesis progression to ESCC, as shown in Fig. 2 and Fig. 3. The evidence today indicates that esophageal microbiota composition can predict the outcome of EC and help identify patients at risk of EC.

## Role of chronic inflammation in the development of EC

There is ample evidence indicating that the inflammatory microenvironment plays a role in carcinogenesis in many cancers by generating a different form of reactive oxygen species. As a result, “inflammation” was among one of the seven originally envisaged hallmarks of cancer, joining the other six—cancer cells’ ability to multiply, angiogenesis, immortality, resistance to inhibitory signals and apoptosis, and the ability to spread [36]. In GERD and Barrett’s esophagus, persistent gastric acid and bile salt exposure can cause chronic inflammation and esophageal injury. Chronic inflammation leads to increased secretion of pro-inflammatory cytokines, chemokines, prostaglandins, and ROS. These mediators of inflammation induce cell growth and invasion, mutagenesis, and angiogenesis. Furthermore, upon persistent stimulation, the inflammatory mediators lead to transformation and initiation of tumor formation [107]. In addition, these inflammatory factors can also potentially suppress immune function, thereby promoting the risk of cancer development in inflamed regions [108]. During tissue damage, a host response is generated by a complex network of cellular signals that infiltrate the damaged area with immune cells such as macrophages, lymphocytes, dendritic cells, and neutrophils, which starts the healing process. Acute inflammatory factors persist, which results in a state of chronic inflammation. Additionally, chronic inflammation leads to tumor development by forming a local microenvironment that promotes neoplastic transformation and cancer progression by driving mutagenesis. The inflammatory microenvironment can induce the breakage of the basement membrane, thus causing invasion and migration of tumor cells [107] (Fig. 2B).

### Cause/ molecular basis of inflammation

The primary mechanism by which inflammation causes esophageal carcinogenesis is through the constitutive activation of inflammatory signaling pathways, leading to downstream activation of genes involved in tumor growth and survival [109]. One of the main pathways involved in EC is the interleukin-6/STAT3 signaling pathway, which is upregulated in several other cancers [110]. Under normal physiological conditions, the IL-6/STAT3 pathway enables cells to survive toxic inflammatory conditions. The cytokine, IL-6 signals by associating its receptor (IL-6Rα) with gp130 and induces downstream recruitment and activation of several molecules (SHP2, Ras-mitogen-activated protein kinase, and phosphatidylinositol 3 kinase) and the STAT1 and STAT3 transcription factors [111]. The IL-6/STAT3 pathway allows cells to survive in a highly toxic inflammatory environment induced by the immune system to kill pathogens in normal physiological conditions. However, this pathway is hijacked by neoplastic cells during carcinogenesis, thus promoting growth, survival, angiogenesis, and metastasis [112]. Furthermore, there is constitutive activation of STAT3 signaling in cancer that inhibits both apoptosis and antitumor immunity [113, 114] (Fig. 2A).

Several studies have found that cell proliferation and apoptotic resistance in BE and EAC correlated with increased epithelial IL-6/STAT3 activity. In addition, increased serum IL-6 was associated with progression from BE to EAC [115] and with a poor prognosis in ESCC patients receiving neoadjuvant chemoradiotherapy. Additionally, immunohistochemistry analysis revealed that the frequency of IL-6 in EC tissues was much higher than in non-malignant epithelium, and its expression was linked to the development of distant metastasis [116, 117]. Studies in mouse models and human samples show that this pathway is induced due to exposure to bile acid and low pH in the esophagus [23, 118]. Mechanistically, it leads to tumorigenesis as IL-6 drives expansion of pro-tumorigenic MDSCs [119], whereas STAT3 activation leads to the production of anti-apoptotic molecules like myeloid cell differentiation protein-1 (Mcl-1) [120].

The pro-inflammatory transcription factor NF-κB regulates cellular processes like survival, apoptosis, proliferation, and cytokine production. Upon stimulation by factors such as oxidative or inflammatory stimuli, or radiation, it translocates to the nucleus and subsequently activates transcription of genes involved in tumorigenesis, hypoxia, immune evasion, and treatment resistance [121, 122]. NF-κB is overexpressed in both EAC and ESCC, and its activation is considered a critical link between an inflammatory microenvironment and cancer development. Although rarely activated in reflux esophagitis, NF-κB activation is increased in BE and EAC, which suggests that it may be a marker of metaplasia–dysplasia–adenocarcinoma progression in addition to its role as an inflammatory marker. Additionally, in esophagitis and Barrett's epithelium, the levels of pro-inflammatory cytokines IL-8 and IL-1β are high, and in adenocarcinoma, it is dramatically enhanced. However, only patients with adenocarcinoma showed a link between NF-κB activation and cytokine overexpression. This suggests that blocking the NF-kB /pro-inflammatory cytokine pathway could be a key target for future chemoprevention strategies [86]. Reportedly, the expression of NF-κB in EAC samples correlated with the stage of the disease [84], and its expression was induced by acid and bile in experimental models [123]. IL-8 and IL-1β are the two main downstream effectors of NF-kB that play a role in esophageal carcinoma. IL-8 plays a role in angiogenesis, cell survival, migration, metastasis, and immune cell infiltration [95, 124]. Whereas IL-1β is secreted in large quantities by the tumor sites, promoting invasiveness, stemness, and immune suppression [125, 126]. Importantly, both STAT3 and NF-kB pathways interact in a complex and interdependent manner. Amplification of signal is achieved by maintenance of a continuous positive feedback loop established between NF-kB/IL-6/STAT3. Furthermore, several downstream effector molecules are common between STAT3 and NF-kB [127] (Fig. 2A). This redundant nature of molecular interactions imparts plasticity to cancer cells in the presence of inhibitors that target any of these pathways, ensuring their survival [128].

Under normal conditions, TGF-β1, an anti-inflammatory cytokine acts as a tumor-suppressor; however, it is linked with tumorigenesis in the abnormal microenvironment. In the cases of Barrett's adenocarcinoma, it was found that relative expression of TGF-β1was significantly more in tumor tissue when compared with squamous epithelium. Furthermore, its overexpression is associated with reduced survival [126]. TGF-β1 signalling is activated by phosphorylation of the intracellular signalling components Smad2 and Smad3 via type I and II transmembrane serine/threonine kinase receptors. This complex is accompanied by Smad4 [129]. After translocating to the nucleus, there is an activation of target genes, allowing TGF- β1 to act as a negative growth factor. During different stages of Barrett’s-metaplasia-dysplasia, the responsiveness of TGF-β1 is reduced due to abnormalities in the signaling pathway [129]. The epidemiologic relationship between the low risk of EC and the use of NSAIDs has stimulated research into the inflammation and carcinogenesis-related expression of cyclooxygenase-2 [130]. Exposure to bile acid results in significant upregulation of COX-2 in esophageal tissue. COX-2 regulates various processes like angiogenesis cell proliferation changes in the invasion properties of the cancer cell. In Barrett’s-associated EAC, COX-2 is linked to higher lymph node metastases and decreased survival [131].

Under normal conditions, ROS is constantly generated during T cell activation or oxidative phosphorylation in the mitochondria. In BE, high levels of ROS are seen associated with tumor initiation by inducing DSBs and tumour growth [132]. When benign Barrett’s epithelial cell line is exposed to acid, there is a generation of ROS and a time-dependent increase in the levels of phosphoH2AX, a double-strand DNA break marker [133]. The antioxidant defence comprising superoxide dismutase (SOD) and the glutathione redox system are considered protective mechanisms against oxidative stress. As expected in BE, it has been shown that the levels of SOD and glutathione are lower than in normal esophageal mucosa [134]. The tumor core is a site associated with the hypoxic tumor microenvironment. Hypoxia results in stimulating hypoxia-inducible factors (HIF-1 and -2), and subsequent reoxygenation can cause considerable oxidative stress via the generation of nitric oxide, ROS, and H_2_O_2_ [135]. HIF-1α is upregulated in BE and correlates with the inflammation, but since it is not upregulated in dysplasia or neoplasia, it is thought to be an initial event to neoplasia and inflammation [136]. On the other hand, HIF-2 α is upregulated in dysplasia and even more so in EAC, but not in BE, implying that it is active later in the neoplastic process [137]. There is enough scientific evidence to indicate that hypoxia is one of the co-factors which aggravate inflammation in the TME.

The tumor hallmarks associated with angiogenesis are also altered by inflammation which is an important driver of oncogenesis. In the esophagus during the metaplasia to cancer sequential events, plasmacytoid and myeloid dendritic cells are recruited which upon stimulation by TNFα or TGF-α causes metaplastic cells in BE to release VEGF, which can encourage nearby endothelial cell development by phosphorylating beta-catenin [138]. Macrophages are another source of VEGF. In EAC, macrophages are more numerous, and they also secrete matricular protein like matrix metalloproteinase-12 (MMP-12), which increases from BE to EAC. In comparison to reflux esophagitis, inflammation at the site of metaplasia is characterized by an increase in Th2 effector cells, as well as Th1 effector cells, supporting the concept that distinct esophageal immune responses may influence disease progression [139]. The ECM is altered to allow immune cells to infiltrate. Matricellular proteins are elevated in both BE and EAC. A secreted acidic protein high in cysteine has anti-adhesive and anti-proliferative properties and can change the cell cycle and remodel the matrix [140]. It is assumed that overexpression of anti-MMP protein in the tumor microenvironment can inhibit tumor growth. Together, these studies conclude that inflammation alters the TME and facilitate disease progression. The molecular basis of inflammation and how this inflammation is associated with the development of EC are represented in Fig. 2.

## Cross-talk between inflammation and microbiota

Under normal physiological conditions, there exists a state of homeostasis between the microbiota and the immune system at the esophageal epithelium. The wide range of esophageal bacteria regulates inflammation, immunity, and metabolism [60] (Fig. 3). However, “dysbiosis,” that is, any loss of physiological balance in the microbial composition, can lead to various pathological conditions. Interestingly, dysbiosis has been reported to be a common effector in pathways implicated in cancer [141, 142]. Several factors such as hormonal imbalance, dietary compounds, toxins, and antibiotics can lead to dysbiosis. The cross-talk between the microbiota and stomach/ esophageal mucosal immune cells is complex and critical and regulated by an intricate network of cytokines produced by immune cells [143] (Fig. 3). In the case of esophageal/gastric dysbiosis, the immune response to prevent bacterial growth may trigger the process of oncogenesis. The profiling of the gastroesophageal microbial community has shown that dysbiosis is linked with EC [144, 145]. The commensal bacteria play a critical role in the functioning of the adaptive immune system, and conversely, the development of the microbiota is regulated by the host immune system. This was confirmed by a study on germ-free animals that lack intestinal microbiota [146]. These mice have impaired development of the innate immune system and the adaptive immune system. Their intestinal epithelial cells show a reduced expression of TLRs and MHC II and a low number of CD4^+^T-cells. Furthermore, they have decreased IgA and IgG levels in the serum, which along with gut dysbiosis, may lead to gluten-sensitive enteropathies [146]. A study indicates that commensal bacteria colonization can correct the Th2 type allergic response in germ-free mice [147].

In a state of equilibrium, the innate immune cells, through TLR, can recognize foreign antigens such as LPS, peptidoglycan, or flagellin, and can signal via the MyD88 (myeloid differentiation primary response protein)-dependent pathway to induce innate immune cell response [148]. Furthermore, local immunity can be influenced by the bacterial metabolites through IgA production by plasma cells to enhance immune response. The maturation of antigen-presenting cells such as DCs is induced by the PAMPs. Upon activation, the DCs can interact and stimulate naive T-cells to form CD4^+^T–cells, and DCs may also directly stimulate CD8^+^T -cells [149]. The adaptive immunity at distant sites can be induced by effector molecules like cytokines and interferons produced by innate immune cells via TLR recognition of microbial peptides and downstream signaling [149]. Furthermore, after priming by antigen-presenting DCs, the Tregs and Th17 cells can circulate systemically to induce immune responses to specific antigens at distant sites [150]. Therefore, impaired local and systemic immune responses can result from a dysbiotic microbial composition which further leads to activation of inflammatory pathways whose hyperactivation leads to accumulation of DSBs at the cellular level, which give rise to EC tumor.

## Inflammation and microbiota mediated pathways as avenues for therapeutic interventions

As discussed earlier, it is well established that the IL-6/STAT3 pathway is the prominent pathway that induces esophageal oncogenesis and is a potential target for therapy in EC. For example, IL-6 inhibition in ESCC cell lines using small interfering RNA-mediated led to enhanced chemosensitivity and increased cell death, decreased angiogenesis and less epithelial-to-mesenchymal transition (EMT) [117, 151]. Furthermore, inhibiting STAT3 signaling by small-molecule stattic radio sensitized ESCC cells in vivo, especially under hypoxia. Moreover, stattic inhibited STAT3 activation and downregulated HIF-1α and VEGF expression and is a potential adjuvant for the radiotherapy in ESCC [43]. Stattic also induced apoptosis in BE and EAC cells and restored chemo- and radiosensitivity [43, 152].

Like STAT3 inhibition, blocking NF-kB sensitizes the esophageal cancer cells to paclitaxel and 5-fluorouracil [153, 154]. Given that IL-6 and IL-8 expression induced by acid may lead to mucosal injury in GERD, thus inhibition of the pro-inflammatory cytokine pathways like NF-kB may act as an important therapeutic target for treating esophageal inflammation. A plant-derived anti-NF-kB compound, curcumin, inhibits the esophageal inflammatory response to bile and acid in BE and EAC [154] (Table 1). IL-1β, secreted from the tumor microenvironment or the tumor cells, stimulates inflammation that induces invasiveness. Inhibition of IL-1β reduces tumor invasiveness, thus pointing to its utility in cancer therapy. COX-2 inhibitors have also shown therapeutic potential in esophageal cancer by targeting inflammatory pathways. Several studies have demonstrated that both selective and nonselective COX-2 inhibitors reduce inflammation and inhibit cell growth, and at the same time, induce apoptosis in BE and EAC [155, 156]. Furthermore, chronic intake of non-steroidal anti-inflammatory drugs is linked with a decreased incidence of EAC.

**Table 1:** Inflammation and microbiota mediated pathways as avenues for therapeutic interventions

Type of esophageal disease	Pathway targeted	Therapeutic intervention	Cell line/ mouse model/organoid	Outcome of intervention	Reference
ESCC	IL-6/STAT3	IL-6 specific siRNA	Human EC cell line TE13& KYSE170	Suppression of cisplatin induced cytotoxicity	[151]
ESCC	IL-6/STAT3	Human IL6 shRNA	Human EC SS cell line CE81T	Increased cell death, less epithelial-mesenchymal transition and attenuated STAT3 activation, attenuated angiogenesis	[117]
ESCC	STAT3	Small-molecule inhibitor, Sttatic	ECA109, TE13,KYSE150	Confers radiosensitivity in ESCC cells in vito& in vivo	[43]
BE & EAC	STAT3	Honokiol (polyphenol)	BE and EAC cell lines	Necrosis and apoptosis in transformed BE and EAC cells	[152]
EC	NF-κB	BAY11-0782 ( NF- κB inhibitor)	EC cell line SKGT5, HCE4, and TE2	Sensitized cancel cells to paclitaxel (induction of apoptosis)	[153]
GERD	NF-κB	Curcumin, an inhibitor of NF-κB (SN-50)	Human esophageal epithelial (HET-1A) cells	Curcumin inhibits esophageal activation in response to acid(reduced esophageal inflammation)	[154]
EAC	COX-2	Selective COX-2 inhibitor (NS-398)	EAC cell lines (FLO, SEG1, BIC1)	Decreases cell growth, Increases apoptosis in Barretts associated adenocarcinoma	[155]
EC	COX-2	Selective COX inhibitor (Coxibs)		Chronic exposure to coxibs was associated with a significant risk reduction for EC	[156]
ESCC	COX-2	Selective COX-2 inhibitor (NS-398)	Human ESCC cell lines (KYSE450&KYSE510)	Suppressed production of prostaglandin E2(PGE2), induced cell growth inhibition, cell cycle arrest	[157]
EC	STAT3 and MEK/ERK signaling	Small-molecule inhibitors of STAT3 and MEK1/2 signaling	3D organotypic model of EC	Suppression of tumorigenesis in the 3D organotypic model of EC	[158]
EAC	Effect of alteration of microbiome through antibiotics on development of EAC	Penicillin G and streptomycin	Surgical rat model for EAC	Alteration of microbiome does not affect The incidence of EAC	[73]
Eosinophilic esophagitis		Lactococcus lactis NCC 2287	Murine model of EoE	Improves esophageal inflammation	[159]

In ESCC, COX-2 inhibition leads to decreased cell proliferation, prostaglandin E(2) production, and overall tumor progression in vitro and in vivo [157] (Table 1). By delineating the mechanisms and the role of the microbiome in the development and progression of EC, research groups are working towards developing novel therapeutics to treat or prevent EC by altering the microbiota composition. This can be achieved by using antibiotics, probiotics, prebiotics, or microbiota transplants. Interestingly, the ability of the microbiota to modulate the toxicity and efficacy of chemotherapy is also being investigated [60]. Reportedly, the microbiota and immune system have enhanced the efficacy of oxaliplatin, an anticancer drug that treats EC [60].

There is relatively little research so far on the effects of probiotics on the esophageal microbiome and how they can aid in preventing EC oncogenesis. Intriguingly, since specific bacteria are implicated in the development of esophageal diseases, like EC, theoretically, therapy aimed at altering the esophageal microbiome could potentially lower risk or improve disease outcomes in EC. In a study on the surgical rat model for EAC, rats were given penicillin G and streptomycin; however, they showed a non-significant reduction in EAC development [73]. There is enough evidence indicating that microbiota and the immune system closely interact, raising the possibility that the microbiota can affect the host’s responsiveness to immunotherapy [160]. To date, there has been no study that has evaluated the effect of probiotics on the inflammatory disease of the esophagus. Eosinophilic esophagitis (EoE) is a severe inflammatory disease of the esophagus in which there is eosinophilic infiltration into the esophageal tissue. A study has identified a probiotic, lactis NCC 2287, which improves esophageal inflammation in experimental EoE [159]. This effect is strain-specific and depends on the timing and duration of bacterial supplementation. Furthermore, phase I clinical trial is underway to assess the safety of a defined mixture of bacterial species administered orally along with immune checkpoint inhibitors (NCT03686202). Another phase I study assesses the tolerability and preliminary efficacy of a microbial cocktail given with immunotherapy in melanoma patients (NCT03817125). Zaharuddin et al. have demonstrated a marked reduction of circulating pro-inflammatory cytokines such as IL-6, tumor necrosis factor–α (TNF-α), IL-17A, IL-17C, and IL-22 in colorectal cancer patients by treatment with a postoperative probiotic mix [161]. Furthermore, Li et al. have shown that a probiotic mix administered to mice after subcutaneous tumor inoculation affects the progression of hepatocellular carcinoma cell growth in mice in a manner identical to cisplatin treatment [162]. Another strategy for modulating the microbiota is through fecal microbiota transplantation (FMT), which involves the transplantation of liquefied and filtered stools of a healthy donor to recipients [163]. Riquelme et al. have utilized FMT in pancreatic cancer patients and shown the ability of gut microbiota to alter the local tumor microbial composition and subsequently modulate the tumor growth response. The group which received the FMT from long-term survivor pancreatic cancer patients had lower oncogenic signatures in comparison to those who received the FMT from short-term survivor pancreatic cancer patients [164]. Collectively, the research suggests that altering the microbiota could shift the inflammatory and immune response towards the anticarcinogenic phenotype and prevent cancer progression.

Since there exists a close relationship between the oral and esophageal microbiome, another potential approach being explored is to alter the oral microbiome. Table 1 lists different dysregulated pathways in EC and how excessive dependence of EC cells on these pathways can be used to develop new therapeutic options.

## Conclusion and Future perspectives

One of the key mediators of inflammatory and neoplastic conditions of the gastrointestinal tract has been identified to be the microbiome. Although with the growing advancement in microbial 16S rRNA sequencing and computational analysis, we have better understood the microbial composition and its role in altering the host immune system and modulating the cancer immunotherapy treatment [165, 166]. However, its role in the pathogenesis of esophageal diseases such as BE, EC, and EoE remains unexplored.

One major challenge is to find new methodologies to evaluate the precise role that diet and medications (antibiotics, PPIs) play in influencing gastroesophageal tumorigenesis and its treatment. Several observational and cross-sectional studies have attempted to characterize the microbial composition implicated in esophageal oncogenesis but have been unable to establish the causal association between the two. Therefore, more mechanistic studies need to be undertaken to determine the roles of specific bacteria in the esophagus and identify novel therapeutic approaches. In this context, the development of animal and organoid models for esophageal cancer can be useful, as they mimic the molecular heterogeneity of clinical EC [167, 168]. This is critical since 2D cell lines lack the genomic characterization of primary cell lines [169]. The genetically engineered mouse models lack well-defined genetic drivers, such as the APC gene in the colon. In a mouse model of BE, a study demonstrated through microbiome transplants that a high-fat diet–induced dysplasia by modulating the esophageal microenvironment and gut microbiome, thereby causing inflammation [170]. Therefore, studies like these are critical in delineating the function of microbiota in the esophageal microenvironment. The difficulty of collecting esophageal samples is another potential limitation, which can be overcome by using non-endoscopic collection methods such as the *Cytosponge*—a mesh contained in a capsule and attached to a string that yields ten times more microbial DNA than endoscopic techniques [74]. Such minimally invasive screening tools will enable screening of patients at high risk for developing various esophageal conditions. Future studies of the microbiome and EC should also consider oral health, given the mounting evidence of an association between the oral pathogen *Porphyromonas gingivalis* and ESCC [76, 171]. Additionally, a clear association between alterations in the esophageal microbiome and the development of dysplasia could provide an important new opportunity for esophageal cancer screening [71]. The etiology of EC may involve infection with multiple bacterial species rather than some prominent and well-studied bacteria such as the *F. nucleatum* and *Porphyromonas gingivalis*. Thus, it is critical to assess if these bacteria are the lone drivers of tumorigenesis by taking into account the entire bacterial community and their mutual interaction as a whole. Furthermore, rather than just identifying bacterial species, it is more important to delineate their functional roles by using high throughput global profiling approaches such as metabolomics, proteomics, and metatranscriptomics to delineate differential functional profiles of the microbiome linked with EC [172]. Thus, understanding the diverse pathway through which the microbiota leads to esophageal carcinogenesis will open new avenues for diagnosing, preventing, and treating EC.

## Abbreviations

EC: Esophageal cancer
ESCC: Esophageal squamous cell carcinoma
EAC: Esophageal adenocarcinoma
GERD: Gastroesophageal reflux disease
NERD: Non-erosive reflux disease
BE: Barret’s esophagus
ROS: Reactive oxygen species
NOS: Nitric oxide synthase
GI: Gastrointestinal
TLR-4: Toll-like receptor-4
HER2: Human epidermal growth factor receptor 2
EGFR: Epidermal growth factor receptor
PI3k/mTOR: Phosphoinositide 3-kinase/mammalian target of rapamycin
TME: Microenvironment
CAFs: Cancer-associated fibroblasts
TAMs: Tumor-associated macrophages
Treg: Regulatory T
MDSCs: Myeloid-derived suppressor cells
DCs: Dendritic cells
CRT: Chemoradiation therapy
HIF-1α: Hypoxia-inducible factor-1α
VEGF: Vascular endothelial growth factor
PCR: Polymerase chain reaction
PPIs: Proton pump inhibitors
LPS: Lipopolysaccharide
IL: Interleukin
TNF: Tumor necrosis factor
PAMPs: Pathogen-associated molecular patterns
COX-2: Cyclooxygenase-2
MSK: Mitogen and stress-activated protein kinase
MAPK: Mitogen-activated protein kinase
TLR-2: Toll-like receptor-2
TLR-9: Toll-like receptor-9
iNOS: Inducible Nitric Oxide Synthase
MMP: Matrix metalloproteinase
NLRP3: Nod-like receptor protein 3
TNFRs: Tumor necrosis factor receptors
DSBs: Double stranded breaks
SOD: Superoxide dismutase
MMP-12: Matrix metalloproteinase-12
ECM: Extracellular matrix
EMT: Epithelial-to-mesenchymal transition
EoE: Eosinophilic esophagiti
